# sMR and PTX3 levels associate with COVID-19 outcome and survival but not with Long COVID

**DOI:** 10.1016/j.isci.2024.110162

**Published:** 2024-06-01

**Authors:** Lisa Hurler, Federica Mescia, Laura Bergamaschi, Cambridge Institute of Therapeutic Immunology and Infectious Disease-National Institute of Health Research (CITIID-NIHR) COVID BioResource Collaboration, Erika Kajdácsi, György Sinkovits, László Cervenak, Zoltán Prohászka, Paul A. Lyons, Erik J.M. Toonen

**Affiliations:** 1 Department of Internal Medicine and Haematology, Semmelweis University, Budapest, Hungary; 2 Cambridge Institute of Therapeutic Immunology and Infectious Disease, Jeffrey Cheah Biomedical Centre, University of Cambridge, Cambridge CB2 0AW, UK; 3 Department of Medicine, University of Cambridge, Addenbrooke’s Hospital, Cambridge CB2 0QQ, UK; 4 Research Group for Immunology and Haematology, Semmelweis University - Eötvös Loránd Research Network (Office for Supported Research Groups), Budapest, Hungary; 5 Research and Development Department, Hycult Biotech, Uden, the Netherlands

**Keywords:** Molecular biology, Virology

## Abstract

Biomarkers for monitoring COVID-19 disease course are lacking. Study aim was to identify biomarkers associated with disease severity, survival, long-term outcome, and Long COVID. As excessive macrophages activation is a hallmark of COVID-19 and complement activation is key in this, we selected the following proteins involved in these processes: PTX3, C1q, C1-INH, C1s/C1-INH, and sMR. EDTA-plasma concentrations were measured in 215 patients and 47 controls using ELISA. PTX3, sMR, C1-INH, and C1s/C1-INH levels were associated with disease severity. PTX3 and sMR were also associated with survival and long-term immune recovery. Lastly, sMR levels associate with ICU admittance. sMR (AUC 0.85) and PTX3 (AUC 0.78) are good markers for disease severity, especially when used in combination (AUC 0.88). No association between biomarker levels and Long COVID was observed. sMR has not previously been associated with COVID-19 disease severity, ICU admittance or survival and may serve as marker for disease course.

## Highlights

• sMR associates with COVID-19 disease severity, survival, and long-term immune recovery
• sMR has not previously been associated with COVID-19
• sMR has potential as biomarker for severity, especially in combination with PTX3

## Graphical abstract

**Figure:**
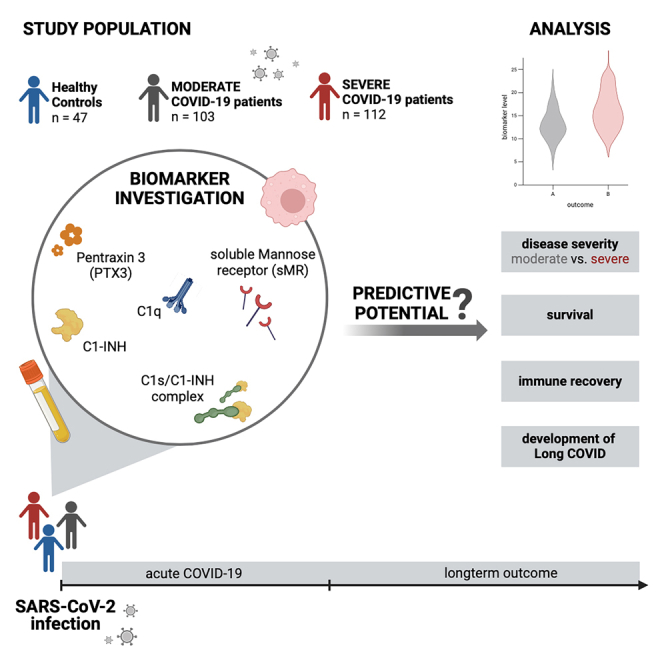

## Introduction

Coronavirus disease 2019 (COVID-19) is a highly infectious respiratory illness caused by the severe acute respiratory syndrome coronavirus-2 (SARS-CoV-2). The high infectivity and severity of the disease resulted in significant morbidity and mortality rates worldwide, with over 774 million confirmed cases and over 7 million deaths as of February 2024.^1^ COVID-19 disease course is unpredictable and patients show symptoms that vary widely, ranging from asymptomatic or mild symptoms to life-threatening conditions such as pneumonia, pulmonary edema, respiratory failure, vascular hyper-permeability, acute respiratory distress syndrome (ARDS), septic shock, multiple organ failure, and death.^2^ In addition, comorbidities such as obesity, diabetes, and cardiovascular disorders further increase the risk of severe COVID-19 outcomes, leading to a more challenging clinical course.^3^^,^^4^ A substantial portion of the individuals infected with SARS-CoV-2 also suffer from long-term multi-organ dysfunction following recovery from acute COVID-19, referred to as Long COVID, post-COVID-19 syndrome or post-acute sequelae of SARS-CoV-2 infection (PASC).^5^^,^^6^

The mechanisms underlying COVID-19, especially Long COVID, remain poorly understood. After infection, the virus starts to replicate and this results in cellular damage, (over)activation of immune cells and sustained release of pro-inflammatory factors due to cell injury and cell death.^7^ The complement system plays a pivotal role in the initial innate immune response to pathogens, including SARS-CoV-2.^8^^,^^9^^,^^10^^,^^11^ Complement activation leads to endothelial damage and production of the anaphylatoxins responsible for the recruitment of leukocytes and release of inflammatory factors.^12^ Indeed, a subset of patients with COVID-19 exhibit hyper-inflammatory responses characterized by excessive activation of myeloid cells, including monocytes, macrophages, and neutrophils, and a plethora of pro-inflammatory cytokines and chemokines.^10^^,^^13^ This excessive and maladaptive systemic inflammatory response could result in several acute clinical pathologies, including lung damage, severe pneumonia, ARDS, and various coagulopathies.^14^^,^^15^^,^^16^ Furthermore, COVID-19 has features reminiscent of macrophage activation syndrome (MAS).^17^^,^^18^ When these aforementioned pathologies are not treated adequately during the acute phase of the disease, possible tissue hypoxia and reduced oxygen exchange can linger for months and may therefore be responsible for the onset of Long COVID.^5^^,^^19^

**Figure 1:**
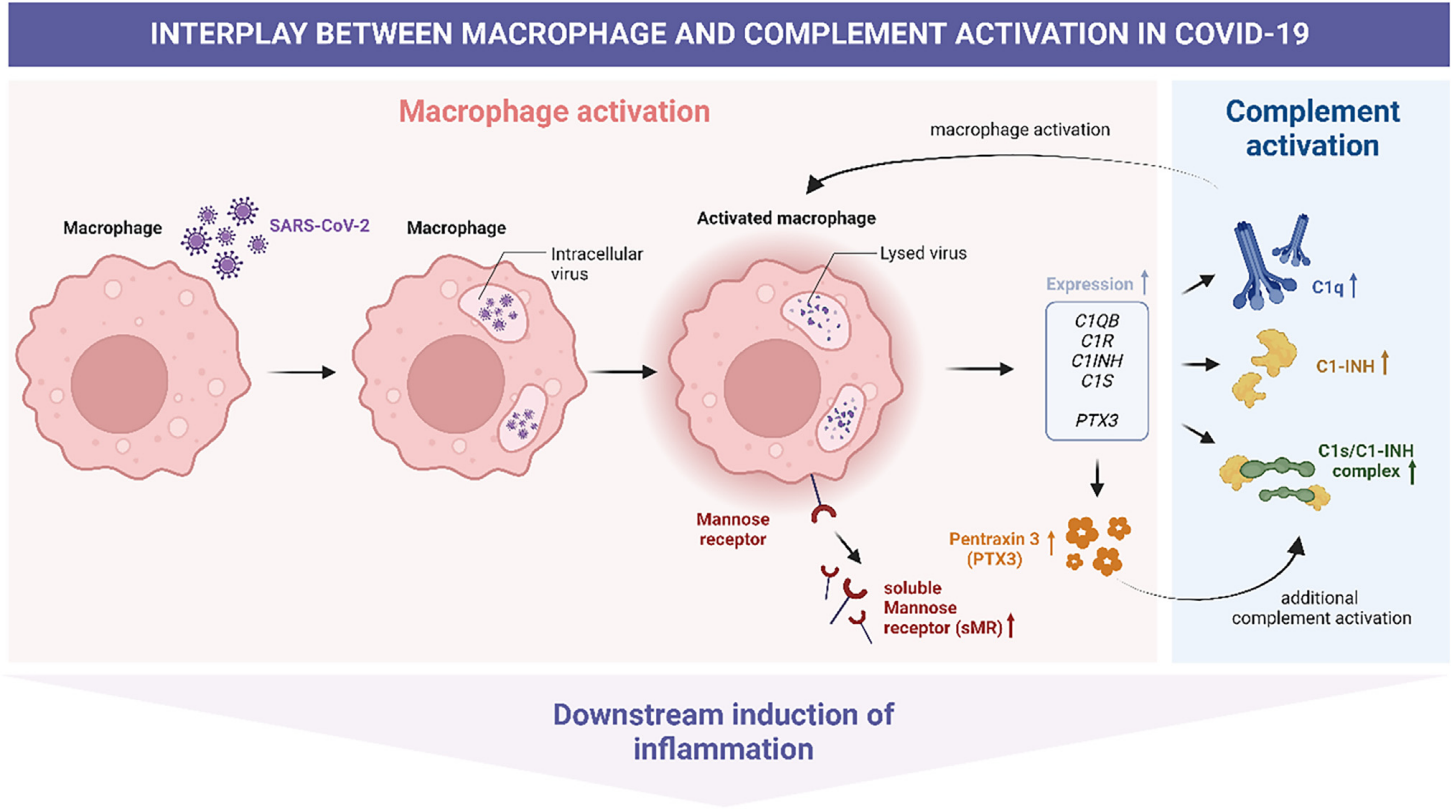
Overview of how the selected biomarkers may interact after SARS-CoV-2-induced macrophage activation leading to complement activation and downstram release of inflammatory factors Figure created with BioRender.com.

Several potential biomarkers have been identified that are associated with COVID-19 disease severity and mortality. These include, among others, increased white blood cell count (neutrophils), decreased lymphocyte (particularly T lymphocytes) and platelet counts, elevated plasma levels of C-reactive protein (CRP), ferritin, interleukin-6 (IL-6), and D-dimer, and complement activation markers.^10^^,^^20^^,^^21^^,^^22^^,^^23^ In addition, also body mass index (BMI) was identified as a potential biomarker for disease severity.^3^ However, clinically validated biomarkers for COVID-19 disease severity, long-term outcome, and Long COVID are lacking, thereby hampering accurate diagnosis and targeted therapies.^24^ Here, we aimed to identify biomarkers that associate with disease severity, survival, long-term outcome, and Long COVID and that have potential as prognostic markers in COVID-19. Given that excessive activation of myeloid cells is strongly associated with maladaptive inflammatory responses in COVID-19 and that complement activation has a key role in this, we focused on proteins involved in these processes. We included proteins previously associated with either the initial inflammatory response in COVID-19 but for which long-term effects are unknown, or with disease severity in other (respiratory) diseases that resembles (aspects of) COVID-19. For complement, pentraxin 3 (PTX3), complement 1q (C1q), C1 inhibitor (C1-INH), and complement C1s/C1-inhibitor complex (C1s/C1-INH) were selected. We also included the soluble form of the mannose receptor C-type 1 (sMR; sCD206), a marker for proinflammatory macrophage activation (Figure 1).^25^ PTX3 is rapidly produced and released by various immune cell types as a response to inflammatory signals.^26^ It binds C1q with high affinity, thereby promoting complement activation.^26^ PTX3 was previously suggested as marker for disease severity and short-term mortality in COVID-19^27^^,^^28^ and also C1q was reported to be involved in this process.^29^ In that light, also C1-INH and C1s/C1-INH complex were selected as they are located directly downstream of PTX3 and C1q and also involved in COVID-19 pathogenesis.^20^^,^^30^^,^^31^^,^^32^ Finally, PTX3 has a profound effect on macrophage priming by inducing mannose receptor C-type 1 (MR) expression.^33^ Given that hyper-inflammatory responses during COVID-19 are characterized by excessive macrophage activation and that sMR is associated with disease severity in community-acquired pneumonia (CAP)^34^^,^^35^ and sepsis,^36^^,^^37^ diseases resembling aspects of COVID-19, also sMR was included.

In the current study, we investigated these markers for potential associations with disease severity, survival, immune recovery, and Long COVID using a well-characterized cohort consisting of 215 COVID-19 patients and 47 controls. For each marker, we compared plasma concentrations between patients and healthy individuals and between COVID-19 patient subgroups stratified by disease severity, survival, immune recovery, and Long COVID.

## Results

### Patient characteristics

A total of 215 COVID-19 patients and 47 healthy controls were included in this study. Baseline characteristics for both groups are summarized in Table 1 and have been reported previously.^38^ The mean age of the patient group was significantly higher than the controls (52.9 ± 17.8 vs. 42.3 ± 15; *p* = 0.0002). No differences were observed in gender distribution between the two groups. Regarding disease severity, 103 patients were assigned to the moderate disease group, consisting of 56 patients (26.0%) not requiring hospitalization and 47 patients (21.9%) who were hospitalized but did not require additional O_2_ or ventilation. The severe group consisted of 112 patients, of which 40 were hospitalized patients requiring O_2_ (18.6%) and 72 were patients admitted to ICU (33.5%). For Long COVID, the total number of patients that responded to the questionnaire asking about persisting symptoms after infection, was 60. Long COVID was observed in 32 out of those 60 patients (53.3%). In total, 19 out of 215 COVID-19 patients (8.8%) died due to the disease or its complications.

**Table 1:** Descriptive statistics of the patient and control cohorts

Variables	COVID-19 patients	Healthy controls	Patients vs. controls
total, n	215	47	–
Male sex, n (%)	118 (54.9)	26 (55.3)	*p* > 0.9999
Mean age ±SD	52.9 ± 17.8	42.3 ± 15.0	*p* = 0.0002
Delay between first symptom and sampling, days median (IQR)	11 (6–31)	–	–
MODERATE cases	n (% of total cases)		
not requiring hospitalization (MILD)	56 (26.0)	–	–
hospitalized, but not requiring O_2_ or ventilation (HOSP)	47 (21.9)	–	–
SEVERE cases			
hospitalized, requiring O_2_ (HOSP+O_2_)	40 (18.6)	–	–
Intensive care unit (ICU)	72 (33.5)	–	–
Disease outcome			
Long COVID^a^, n (%)	32 (53.3)	–	**-**
COVID-19 related death/mortality, n (%)	19 (8.8)	–	**-**
Laboratory findings, median (IQR)			
PTX3 (ng/mL)	2.4 (1.1–6.7)	0.7 (0.4-1.0)	*p* < 0.0001
C1q (ng/mL)	15028 (12228–17900)	13989 (12067–15783)	*p* = 0.0282
C1-INH (mg/mL)	0.266 (0.212-0.322)	0.173 (0.140-0.188)	*p* < 0.0001
C1s/C1-INH complex (ng/mL)	2099 (1516–2826)	2007 (1581–2358)	*p* = 0.2661
sMR (ng/mL)	663 (424–1172)	297 (215–394)	*p* < 0.0001

a The total number of patients who were administered the questionnaire regarding persisting symptoms (Long COVID) was 60.

### Biomarker levels correlate with COVID-19 disease severity

To investigate whether the selected biomarkers were associated with COVID-19 disease course, we compared median plasma concentrations between patients and controls, moderate versus severe disease and patients with or without need for ICU admittance.

**Figure 2:**
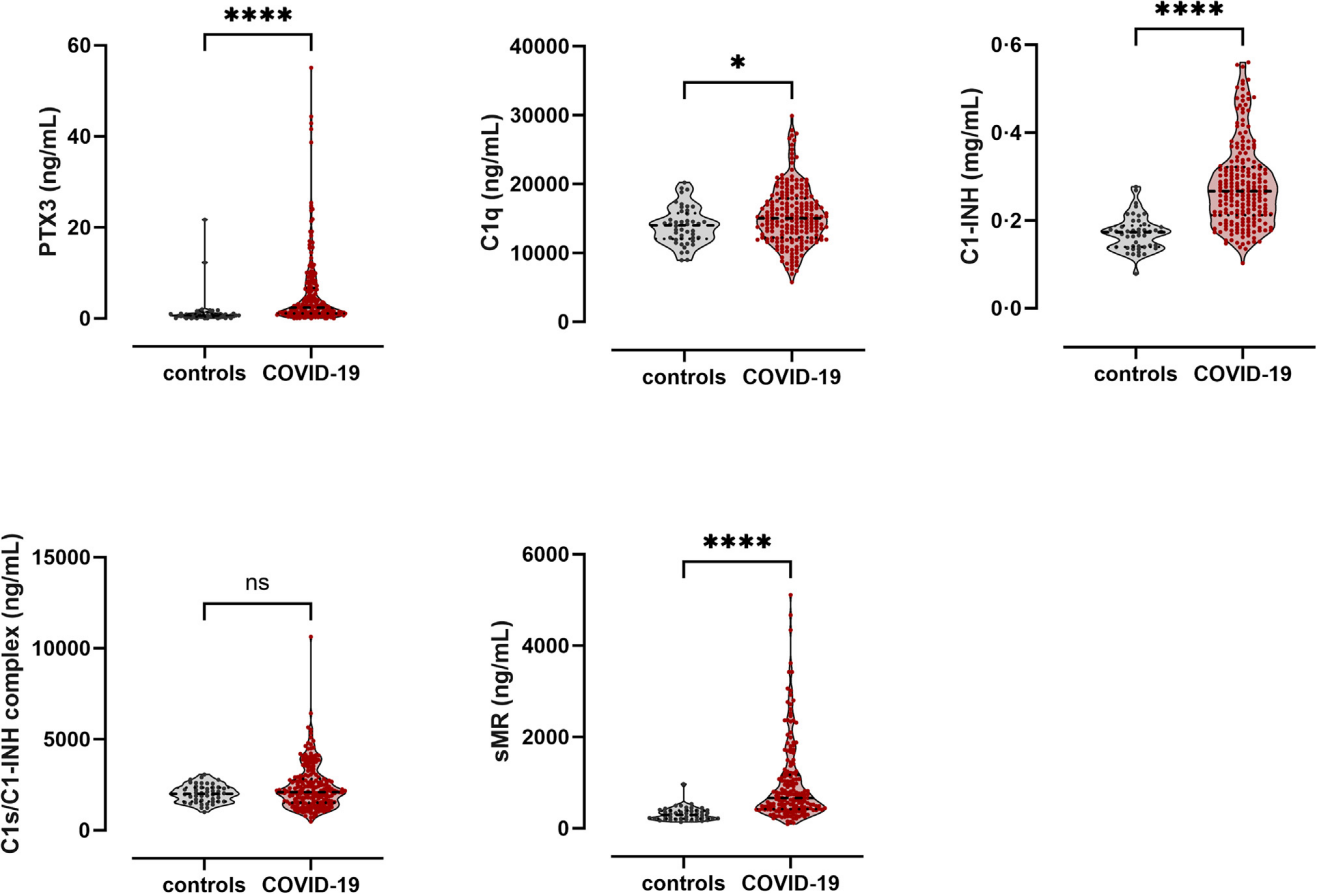
Biomarker plasma levels of COVID-19 patients and healthy controls EDTA-plasma concentrations for all six biomarkers were measured in samples from 215 COVID-19 patients and 47 healthy controls using ELISA. Sampling was done at study entry, soon after infection or symptom onset (day 0). Data are presented as medians and interquartile ranges (IQR). ∗*p* < 0.05, ∗∗*p* < 0.01, ∗∗∗*p* < 0.001, ∗∗∗∗*p* < 0.0001, ns = non-significant.

For all biomarkers except C1s/C1-INH complex, plasma levels were significantly different in samples taken from COVID-19 patients at study entry (soon after infection or symptom onset; 9 (5–18) days; further referred to as day 0) when compared to healthy controls (Table 1; Figure 2). For PTX3, a 3.4-fold increase in plasma levels was observed in patients when compared to controls (*p* < 0.0001). Also the plasma levels for C1q, C1-INH and sMR, were increased in patients, with respective fold inductions of 1.1 (*p* = 0.0282), 1.5 (*p* < 0.0001) and 2.2 (*p* < 0.0001). No changes in levels between patients and controls were observed for C1s/C1-INH complex (*p* = 0.2661).

Next, the moderate versus severe disease patient groups were compared and baseline characteristics for both groups are summarized in Table 2. Mean age of the severe patient group was significantly higher than the moderate group (60.0 ± 14.2 vs. 45.3 ± 17.6; *p* < 0.0001). Regarding gender distribution, the number of male patients was higher in the severe group when compared to the moderate patient group (72.3% vs*.* 35.9%; *p* < 0.0001). As obesity and its associated diseases are known to be risk factors for developing severe disease, also BMI and the number of cases for type 2 diabetes mellitus (T2DM) and coronary artery disease (CAD) were compared between moderate and severe COVID-19 patients. No difference in mean BMI scores was observed between the two disease groups (*p* = 0.0784). For both T2DM and CAD, the number of cases was not higher in the severe group when compared to moderate patients (resp. *p* = 0.2647 and *p* = 0.1022) (Table 2; Figure S1).

**Table 2:** Descriptive statistics of moderate vs. severe COVID-19 cases

Variables	Moderate COVID-19	Severe COVID-19	Moderate vs. severe
total, n	103	112	–
Male sex, n (%)	37 (35.9)	81 (72.3)	*p* < 0.0001
Mean age ±SD	45.3 ± 17.6	60.0 ± 14.2	*p* < 0.0001
Mean BMI ±SD	27.3 ± 5.5	29.1 ± 5.0	*p* = 0.0784
T2DM, n (%)^a^	12 (26.7)	40 (36.7)	*p* = 0.2647
CAD, n (%)^a^	4 (8.9)	22 (20.2)	*p***=** 0.1022

a Info regarding comorbidities only available for 45 moderate and 109 in the severe patients. BMI = body mass index; T2DM = Type 2 diabetes mellitus; CAD = coronary artery disease.

**Figure 3:**
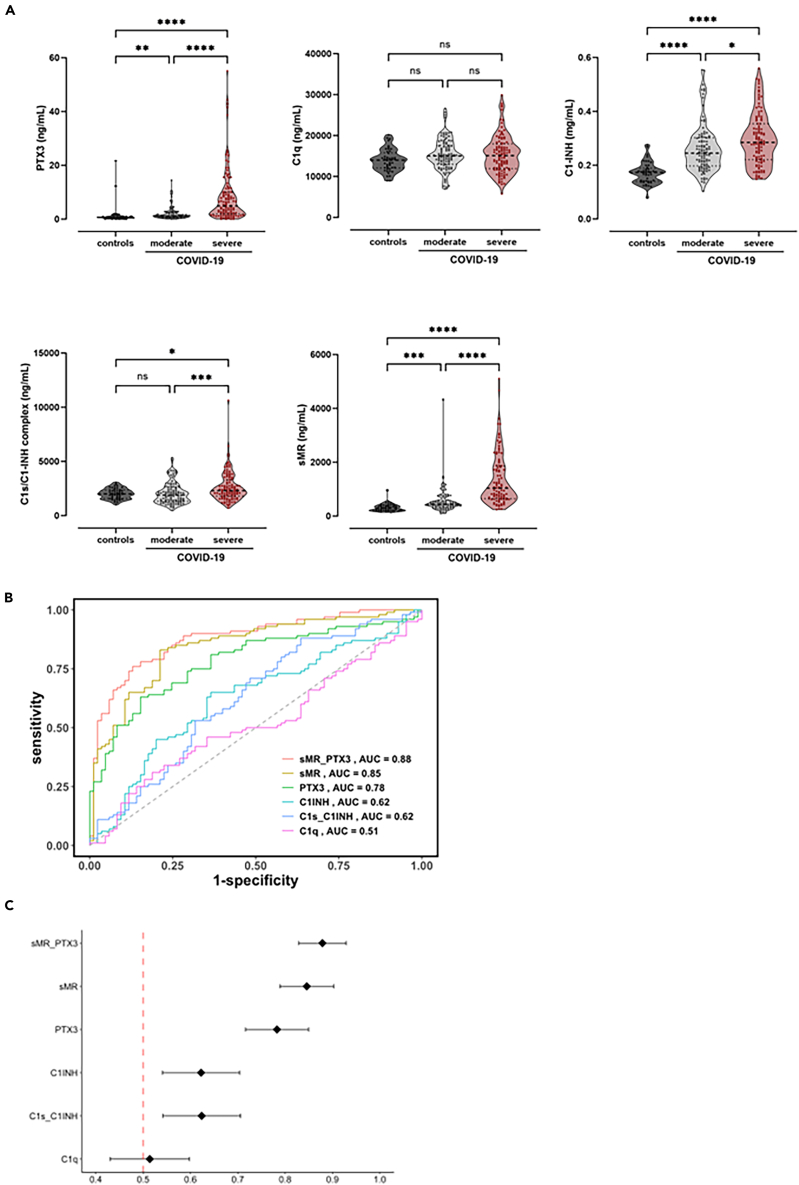
Biomarker plasma levels in healthy controls and COVID-19 patients stratified according disease severity and receiver operating characteristic curves to predict disease severity (A) EDTA-plasma concentrations were measured in samples from COVID-19 patients groups classified as either “moderate” or “severe” disease. Sampling was done at study entry, soon after infection or symptom onset (day 0). (B) Receiver operating characteristic (ROC)-analysis for all biomarkers to illustrate diagnostic potential to distinguish between moderate and severe disease. (C) Area under the ROC curves (AUC) values with 95% confidence intervals were calculated, and biomarkers were ranked according to their capability to distinguish between moderate and severe disease. Data are presented as medians and interquartile ranges (IQR). ∗*p* < 0.05, ∗∗*p* < 0.01, ∗∗∗*p* < 0.001, ∗∗∗∗*p* < 0.0001, ns = non-significant.

Subsequently, we investigated whether biomarker plasma concentrations at day 0 are associated with COVID-19 disease severity by comparing moderate vs. severe disease. Biomarker levels from healthy controls were included as baseline/reference range. Patients with severe disease showed a 4-fold induction in PTX3 plasma levels compared to those with moderate disease (*p* < 0.0001) (Table 3; Figure 3A), confirming previously reported results that PTX3 levels are associated with COVID-19 severity.^27^^,^^28^ In addition, both C1-INH and C1s/C1-INH showed a 1.2-fold increase in plasma concentrations in the severe disease group when compared to moderate (*p* = 0.022 and *p* = 0.0007, respectively), although these increases were less profound compared to PTX3 (Table 3; Figure 3A). Interestingly, sMR plasma concentrations were strongly and significantly increased in patients with severe disease compared to those with moderate disease (2.4-fold induction; *p* < 0.0001) (Table 3; Figure 3A). No difference in plasma concentrations between moderate and severe disease was observed for C1q (*p* > 0.9999).

**Table 3:** Biomarker levels in moderate vs. severe COVID-19 cases and in severe non-ICU vs. ICU patients

Variables	Moderate COVID-19	Severe COVID-19	Moderate vs. severe
PTX3 (ng/mL)	1.2 (0.7-2.6)	4.9 (1.9–10.1)	*p* < 0.0001
C1q (ng/mL)	15028 (12984–17569)	15099 (11916–17986)	*p* = 0.6936
C1-INH (mg/mL)	0.245 (0.197-0.302)	0.285 (0.220-0.354)	*p* = 0.0026
C1s/C1-INH complex (ng/mL)	1903 (1317–2621)	2305 (1795–3292)	*p* = 0.0004
sMR^a^ (ng/mL)	436 (338–567)	1038 (648–1858)	*p* < 0.0001


Variables	Non-ICU patients	ICU patients	Non-ICU vs. ICU
total, n	40	72	
PTX3 (ng/mL)	6.5 (2.0–11.2)	3.7 (1.6–10.1)	*p* = 0.2285
C1q (ng/mL)	13903 (11568–16429)	15865 (12332–18515)	*p* = 0.1197
C1-INH (mg/mL)	0.322 (0.271-0.396)	0.271 (0.209-0.321)	*p* = 0.0043
C1s/C1-INH complex (ng/mL)	2038 (1483–2970)	2397 (2009–3454)	*p* = 0.0779
sMR^b^ (ng/mL)	687 (589–918)	1434 (824–2334)	*p* < 0.0001

Biomarkers levels are depicted in median (IQR). As ICU admittance is confounded by severity, only severe COVID-19 patients are included in analyses comparing ICU vs*.* non-ICU patients.

a Measurement only available for 85 moderate and 100 severe COVID-19 cases.

b Measurement only available for 36 non-ICU and 64 ICU patients of the severe patient group.

Receiver operating characteristic (ROC) curves were plotted to identify markers that show the strongest association with disease severity in this cohort. Area under the ROC curve (AUC) values were calculated, and biomarkers were ranked according to their capability to distinguish between moderate and severe disease (Figures 3B and 3C). The biomarker showing the highest AUC value was sMR (AUC 0.85; *p* < 0.0001), followed by PTX3 (AUC 0.78; *p* < 0.0001), indicating good performance. AUC for both C1-INH and C1s/C1-INH was 0.62 (*p =* 0.0026) and *p* = 0.0004, respectively), indicating moderate/poor performance. For C1q, AUC value was 0.51 (*p =* 0.6928), indicating that this marker is not able to distinguish between moderate and severe disease. Multivariate predictors were generated to investigate whether a combination of sMR and PTX3 perform better when compared to the univariate model, evaluating the performance of single biomarkers. Analysis showed an AUC of 0.88 *(p* < 0.0001) for the sMR_PTX3 combined biomarker set (Figures 3B and 3C), suggesting that this set performs better when compared to the individual biomarkers. Youden’s Index was calculated for all markers to further assess diagnostic performance. For sMR, a value of 62% was calculated, suggesting added value as diagnostic test to distinguish between moderate and severe disease. Also for the sMR_PTX3 combined biomarker set, a value of 62% was calculated. However, the positive likelihood ratio (assessing probability that severe disease will develop) was higher in the combined set (6.271) when compared to sMR and PTX3 individually (3.919 and 4.138, respectively). For all other markers, Youden’s index was <50%. Cut-off concentrations, sensitivity & specificity, likelihood ratio and Youden’s index are outlined in Table 4.

**Table 4:** Biomarkers characteristics, cut-off determination and ranking according to the Youden’s index (J)

Marker	cut-off point	Sensitivity	Specificity	Likelihood ratio^a^	Youden’s index (J)
Comparison of moderate vs. severe disease					
sMR/PTX3 combined (ng/mL)	604.5/3.05	74.0	88.2	6.271	62%
sMR (ng/mL)	604.5	83.0	78.8	3.919	62%
PTX3 (ng/mL)	3.05	64.3	84.5	4.138	49%
C1-INH (mg/mL)	0.273	58.9	68.0	1.839	27%
C1s/C1-INH complex (ng/mL)	1904	72.3	50.5	1.461	23%
C1q (ng/mL)	14048	45.5	65.1	1.303	11%

Biomarker characteristics for prediction disease severity.

a Likelihood ratio was calculated as sensitivity/(1-specificity), i.e., true positivity rate divided by the false positivity rate, indicating the diagnostic value of having a positive test result in supporting a certain condition (moderate disease). For calculation of likelihood ratio positive result was considered in the following way: laboratory parameters below the cut-off were regarded as positive, as these values are supposed to indicate moderate disease, which is the condition in question.

To identify potential interactions underlying COVID-19 disease course, correlation patterns between all measured biomarkers were investigated. In addition, it was investigated whether the levels of the biomarkers investigated here correlated with other (inflammatory) factors known to be associated with COVID-19 severity.^9^^,^^10^ These include: C3a, C3c, soluble TCC, hsCRP, IL1β, IL6, IL10, TNFα, IFNγ, D-dimer, ferritin, BMI, and age (Table S1 and Figure S2). Only weak or moderate correlations were observed, except between sMR and D-dimer levels (Spearman r = 0.77) (Figure S3). Both sMR and D-dimer are able to predict severity and considering them together enhances the predictive ability compared to models considering each individually (delta AIC >2). Note that data regarding these additional parameters were reported previously.^10^

**Figure 4:**
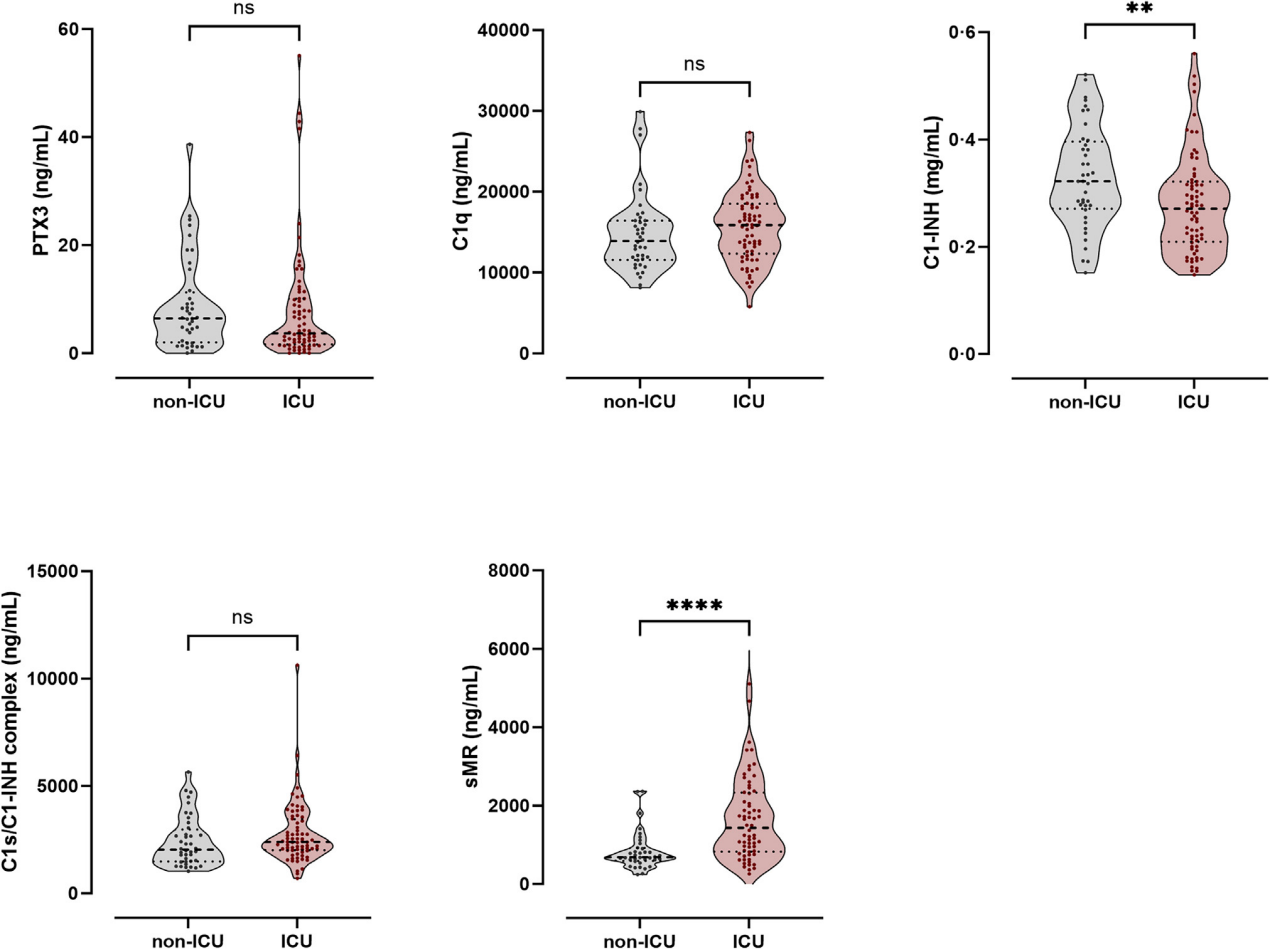
Biomarker levels in severe COVID-19 cases, stratified according to their need of ICU admission EDTA-plasma concentrations at day 0 in COVID-19 patients stratified according to their need of ICU admission. ICU = intensive care unit, non-ICU = not admitted to the intensive care unit. Data are presented as medians and interquartile ranges (IQR). ∗*p* < 0.05, ∗∗*p* < 0.01, ∗∗∗*p* < 0.001, ∗∗∗∗*p* < 0.0001, ns = non-significant.

Additionally, it was investigated whether biomarker concentrations were associated with ICU admittance (Figure 4; Table 3). As ICU admittance is confounded by disease severity, associations between biomarker levels and ICU admittance were only investigated in the severe disease group. For sMR, a 2.1-fold increase in plasma concentrations was observed in patients admitted to the ICU when compared to non-ICU patients (*p* < 0.0001). For C1-INH, ICU patients showed a 0.8-fold decrease in levels when compared to non-ICU (*p* = 0.0043). For the markers PTX3, C1q, and C1s/C1-INH, no differences in levels were observed between severe ICU and non-ICU patients.

Overall, these results show that the biomarkers PTX3, sMR and, to a lesser extent, C1-INH and C1s/C1-INH are associated with disease severity in this COVID-19 patient cohort. AUC values indicate that the combined marker set sMR_PTX3 preforms best to distinguish between moderate and severe disease, followed by sMR and PTX3 as individual markers. Interestingly, the results also show that sMR levels are strongly associated with ICU admittance in severe patients.

### PTX3 and sMR plasma concentrations are associated with mortality in COVID-19

**Figure 5:**
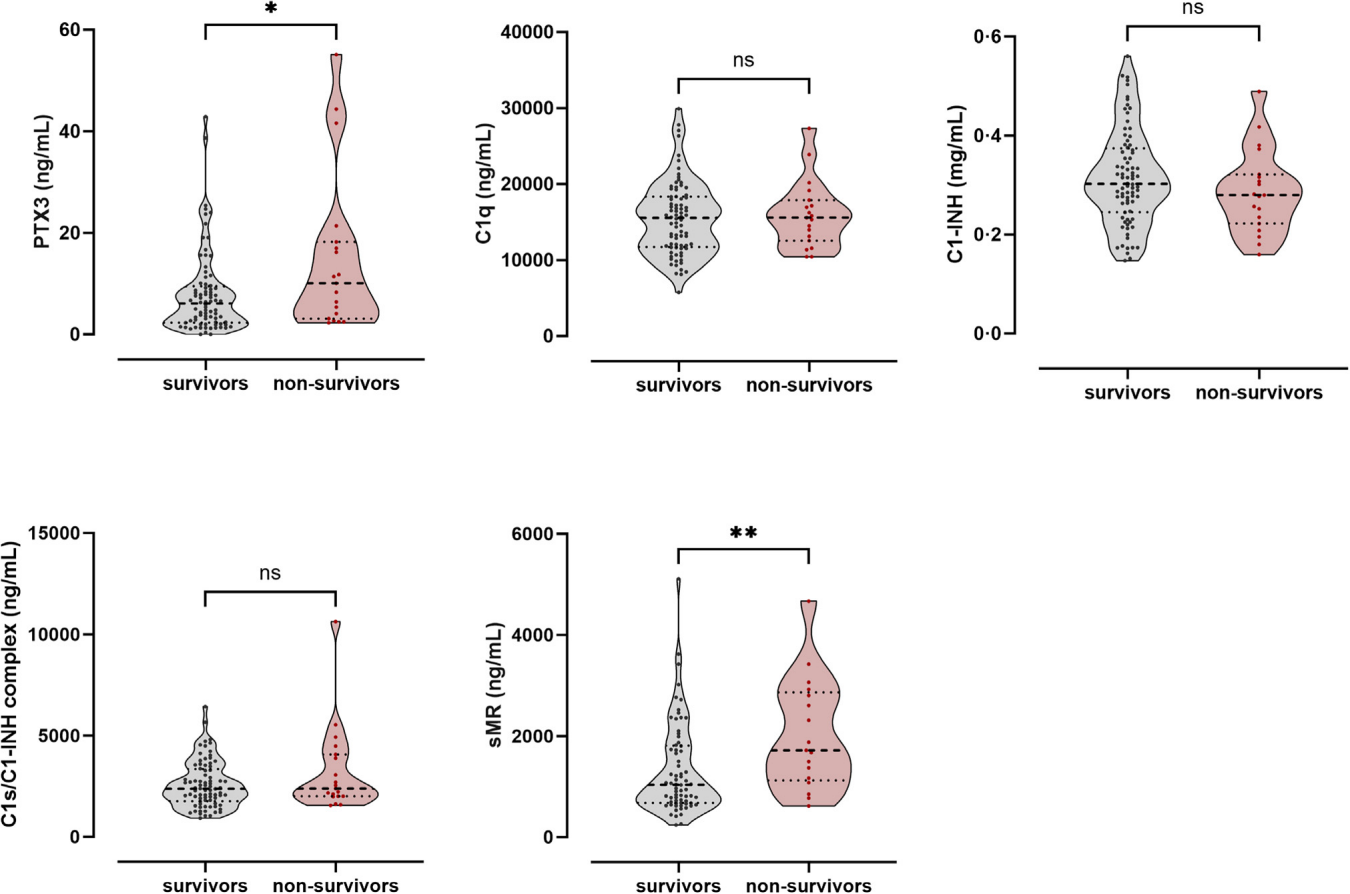
Biomarker levels in severe COVID-19 cases, stratified according to survival EDTA-plasma concentrations at day 0 in severe COVID-19 patients stratified according to survival/non-survival. Data are presented as medians and interquartile ranges (IQR). ∗*p* < 0.05, ∗∗*p* < 0.01, ∗∗∗*p* < 0.001, ∗∗∗∗*p* < 0.0001, ns = non-significant.

All biomarkers were further assessed with regard to their association with survival. Similar to ICU admittance, also mortality is confounded by disease severity. Therefore, associations between biomarker levels and mortality were investigated only in the severe disease group. Both PTX3 and sMR plasma concentrations were 1.7-fold increased in non-survivors when compared to survivors (*p =* 0.0388 and *p* = 0.0070, respectively) in the first few days after infection or symptom onset (day 0). No significant differences in plasma concentrations were observed for the markers C1q, C1-INH, and C1s/C1-INH (Figure 5). These results suggest that PTX3 and sMR plasma concentration during the acute phase of infection are associated with COVID-19 mortality.

### Biomarker plasma concentrations over time after SARS-CoV-2 infection

Biomarker concentrations were investigated during follow-up after SARS-CoV-2 infection. Plasma concentrations in samples collected at day 0 (baseline) and at day 14, 28, and 90 post infection were compared between: moderate and severe patients, ICU and non-ICU patients, and survivors and non-survivors.

With regard to moderate vs. severe disease, plasma concentrations for all biomarkers except C1q were increased in severe patients at day 0, 14, and 28, but normalized after 90 days. C1q concentration in severe patients were increased at day 14 but not at day 0, 28, and 90 (Figure S4). Next, biomarker levels over time were compared between severe patients that were/were not admitted to the ICU. Plasma concentrations for PTX3, C1-INH, and C1s/C1-INH did not differ between ICU and non-ICU patients for all time points. Regarding C1q, ICU patients showed an increase in levels at day 14 (1.4-fold induction; *p* = 0.0003), but not at day 0, 28, and 90 when compared to non-ICU. Levels for sMR strongly increased at day 0 (2.1-fold induction; *p* < 0.0001), day 14 (2.3-fold induction; *p* = 0.0001) and day 28 (3.3-fold induction; *p* = 0.0107), and normalized at day 90 (Figure S5). Lastly, biomarker levels over time were compared between survivors and non-survivors in the severe patient group. PTX3 concentrations showed a trend toward increased levels at day 0 in non-survivors and this increase became significant at day 14 (2.7-fold induction; *p* = 0.0063), but normalized at day 28. Also at day 90, we observed no differences in PTX3 concentrations between survivors and non-survivors (Figure S6). For sMR, a strong increase in concentrations was observed at baseline (1.5-fold induction; *p* = 0.0052 and after 14 days (1.6-fold induction; *p* = 0.0070) in patients who died when compared to those that survived (no data were available for sMR concentrations in non-survivors at day 28). Soluble MR concentrations tended to remain elevated at day 90 in those patients who died and did not seem to normalize. However, due to the low number of patients in the non-survivor group (*n* = 3), this inferred increase did not reach statistical significance (*p* = 0.0636). For the markers C1q, C1-INH, and C1s/C1-INH, no differences in plasma concentrations were observed for any of the time points (Figure S6).

Overall, these results show that plasma concentration for all markers are associated with disease severity during the acute phase of infection. In addition, this longitudinal analysis confirm our initial observations that, during the acute phase, sMR levels are associated with ICU admittance and that both PTX3 and sMR levels are associated with COVID-19 mortality.

### Potential of biomarkers to predict long-term outcome in COVID-19

**Figure 6:**
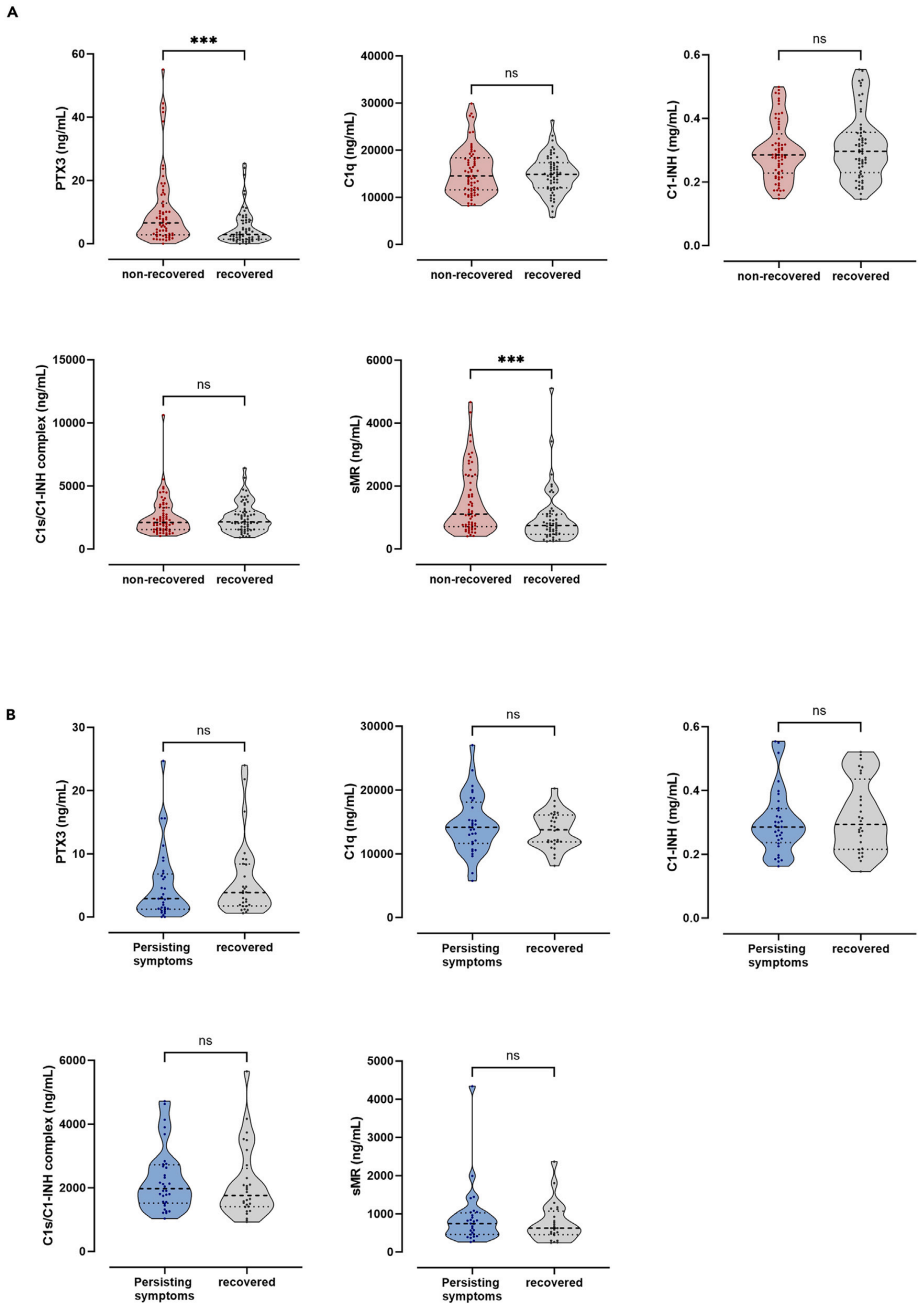
Potential of biomarkers to predict long-term outcome in COVID-19 (A) EDTA-plasma concentrations at day 0 in COVID-19 patients stratified according to immune recovery (yes/no) and (B) Long COVID (yes/no). Data are presented as medians and interquartile ranges (IQR). ∗*p* < 0.05, ∗∗*p* < 0.01, ∗∗∗*p* < 0.001, ∗∗∗∗*p* < 0.0001, ns = non-significant.

Finally, we investigated whether biomarker levels at day 0 were associated with long-term outcome in COVID-19, as defined by immune recovery and the development of Long COVID. In relation to immune recovery, both PTX3 and sMR plasma concentration were strongly increased in non-recovered patients compared to recovered patients (*p* = 0.0004 and *p* = 0.0006, respectively) (Figure 6A). In addition, associations with Long COVID were investigated. For all biomarkers, no differences in plasma levels were observed between patients that recovered and patients that reported persisting symptoms 6–12 months after SARS-CoV-2 infection (Figure 6B).

## Discussion

The clinical course of COVID-19 varies widely and since the beginning of the pandemic, research has been conducted to identify factors that predict disease severity and outcome. Certain clinical conditions and risk factors, such as high age, BMI, and underlying medical conditions like obesity and its associated metabolic diseases are well-known to be associated with increased risk of severe illness.^3^^,^^39^ However, most people infected with SARS-CoV-2, irrespective of underlying risk factors, do not develop severe COVID-19. Presently, no markers are available that are able to predict COVID-19 disease course, comorbidities or the development of Long COVID at disease onset.^40^

In this study, we assessed the association of five soluble biomarkers with disease severity, survival, long-term outcome, and Long COVID in a well-characterized patient cohort. Our results show that plasma levels for PTX3, sMR and, to a lesser extent, C1-INH and C1s/C1-INH, measured soon after infection or symptom onset, are associated with disease severity. ROC curve analysis showed that PTX3 and sMR are promising markers for monitoring disease severity, especially when used as a combined set. None of the markers were associated with the development of Long COVID.

Our results confirm previously reported studies, showing that elevated PTX3 plasma levels are associated with COVID-19 disease severity^41^^,^^42^ and mortality.^27^^,^^43^^,^^44^ The mechanism underlying the association between PTX3 and COVID-19 severity is not fully understood, but recent studies have shed some light on the potential interactions between PTX3 and other proteins that contribute to disease pathogenesis.^42^^,^^44^^,^^45^^,^^46^^,^^47^^,^^48^ One potential mechanism involves the interaction between PTX3 and complement proteins.^45^^,^^49^ Dysregulation of the complement pathway is involved in the pathogenesis of COVID-19^9^^,^^38^^,^^50^^,^^51^^,^^52^ and PTX3 bind to complement proteins such as C1q, MBL, and ficolin-1, thereby regulating/affecting their function.^26^^,^^49^^,^^53^ Other mechanisms involve thromboinflammation,^42^^,^^44^^,^^46^ PTX3-induced leukocyte recruitment and endothelial dysfunction^27^^,^^54^ and regulating cytokine production.^48^

The strongest association between plasma levels, disease severity, survival and long-term immune recovery was observed for sMR. In addition, sMR was the only marker in this set able to distinguish between patients that needed ICU admittance or not, shortly after infection/onset of symptoms. Soluble MR is a marker that had not previously been associated with COVID-19 disease severity and/or survival. Mannose receptor (MR; CD206) is a type I transmembrane C-type lectin receptor predominantly expressed on the surfaces of macrophages, dendritic cells, and endothelial cells. As a cell surface receptor, it plays a role in recognition and uptake of glycoproteins that contain high-mannose oligosaccharides. Its main purpose is to facilitate clearance of glycoproteins, including pathogens such as bacteria and fungi, from the extracellular matrix.^55^ Soluble MR is generated by proteolytic cleavage of the extracellular domain which is then released into the circulation. This shedding from macrophages is mediated by proteases in response to activation via protein C kinase, ATP, ligation of TLR2 or dectin-1 or after infection with e.g., *Candida albicans*, *Aspergillus fumigatus*, *Pneumocystis jirovecii*, and *E. coli*,^56^^,^^57^^,^^58^^,^^59^ but the mechanism is unknown. After shedding, sMR retains its ability to bind ligands and has various functions such as neutralizing pathogens and toxins in the bloodstream and modulation of immune responses.^60^^,^^61^ Increased sMR levels have been shown to be predictive for disease progression and survival in patients infected with tuberculosis,^62^ pneumococci,^63^ or CAP.^34^ In light of this, it should be noted that, while sMR holds potential as a biomarker for assessing COVID-19 severity, it is unlikely to be specific only to COVID-19.

So far, few studies have investigated sMR in relation to COVID-19. Schultheiss and co-workers included sMR as potential biomarker underlying macrophage dysregulation in Long COVID, but reported no differences in levels between the post-COVID-19 group and controls or between individuals with- and without Long COVID.^64^ Also we did not observe an association between sMR levels and Long COVID. However, we did show that sMR levels are associated with severity, ICU admittance, long-term immune recovery, and mortality. Of course, studies differ in experimental design and setup when compared to each other. For instance, Schultheiss et al. did not stratify participants according to disease severity, survival, and immune recovery.^64^ Other differences were time of sampling (8 months vs. 90 days) and inclusion criteria for Long COVID.

Although our results point to sMR as an interesting marker, the mechanism underlying COVID-19 disease progression remains elusive. Several studies showed that the SARS-CoV-2 S-protein is able to bind to other C-type lectin receptors such as DC-SIGN, L-SIGN, and SIGLEC1.^65^^,^^66^^,^^67^ In addition to that, one preprint study shows that SARS-CoV-2 spike protein binds to MR.^68^ It is known that not only expression of MR is upregulated in response to inflammatory stimuli, but also receptor shedding.^61^ It is possible that this is also the case in response to SARS-CoV-2 infection. After upregulation and shedding from the cell surface, sMR might be able to bind SARS-CoV-2 spike protein and act as a decoy receptor to modulate or downregulate inflammatory responses. On the other hand, sMR is known to enhance macrophage activation and proinflammatory cytokine secretion, leading to excessive inflammation and recruitment of immune cells to the lungs.^69^ Structural/conformational differences or variation in expression and/or shedding may explain dissimilarities in COVID-19 disease outcome between patients.

Also C1-INH and C1s/C1-INH levels were associated with disease severity, although differences between groups were less profound as observed for sMR and PTX3. Although C1-INH is a well-known regulator of the complement cascade,^70^ the role of C1s/C1-INH is less established. However, a few studies were reported showing elevated C1s/C1-INH levels in hereditary angioedema (HAE),^71^^,^^72^ glomerulonephritis,^73^ systemic lupus erythematosus (SLE), and rheumatoid arthritis (RA).^74^ Recently, researchers from our group showed that C1s/C1-INH is increased in multisystem inflammatory syndrome in children (MIS-C), a rare, life-threatening complication of severe SARS-CoV-2 infection.^20^

When investigating Long COVID, none of the investigated markers pointed toward a role in the development or prolongation of Long COVID. As such, we were unable to identify markers to be associated with Long COVID. This is likely due to the complex nature of the condition. Also others have attempted to identify biomarkers for Long COVID,^64^^,^^75^^,^^76^^,^^77^^,^^78^ but were hampered by limited sample sizes, heterogeneity in patient populations, and variations in the timing and type of samples collected.^40^^,^^79^ In addition, lack of consensus on the definition and criteria for Long COVID-19 has made it difficult to compare results across studies.^40^

It is known that inflammatory factors (cytokines, among others), factors such as age and underlying medical conditions, especially obesity and its associated metabolic diseases, are associated with disease outcome.^3^ Indeed, we observed an increase in age in the severe patient group, confirming that “inflammaging” is a risk factor for severity. However, no correlation between age and sMR or PTX3 levels was observed. Also no correlation was observed between levels of sMR and PTX3 and other inflammatory factors (C3a, C3c, soluble TCC, hsCRP, IL1β, IL6, IL10, TNFα, IFNγ, and ferritin), suggesting that these factors sort their effects (at least partly) independent from each other. Our data do not show an increased BMI or a higher incidence of T2DM and/or CAD in the severe COVID-19 group. In addition, biomarker levels did not correlate with BMI. An explanation might be that the effects of obesity-induced low-grade chronic inflammation, locally triggered in the adipose tissue and liver and to believe to drive hyperinflammation in severe COVID-19, are not (yet) reflected in the systemic blood circulation. Mean BMI in the patient groups did not differ significantly from each other (27.3 vs. 29.0; overweight but not obese), suggesting that more homogeneous patient groups (low vs. high BMI) are needed to further investigate whether biomarker levels and BMI are correlated.

### Limitations of this study

This study has several limitations. First, the increased biomarker plasma levels observed in (severe) patients are probably not COVID-19 specific. Many of the abnormalities in plasma levels observed in COVID-19 might also be features of other viral infections as a comparison with an appropriate disease control group is missing. Therefore, it is of utmost importance that the biomarkers investigated here, are validated using independent patient cohorts. Besides, only patients from the first COVID-19 wave were included (between March and July 2020), before vaccines were available. Relevance for monitoring these markers in patients that are vaccinated (or have SARS-CoV-2 antibodies due to previous infections) is currently unknown. It is also unknown how biomarker levels are regulated in response to new SARS-CoV-2 variants that emerged after July 2020. Regarding sample collection, collection of the first sample was done at study entry, soon after infection or symptom onset. It is important to acknowledge that time elapsed since the onset of symptoms may vary between individuals. As a result, it is possible that some of the results observed in this study are due to differences in kinetics, leading to a biased representation of the actual situation. Also with regard to Long-COVID, timing of samples was not perfect. Samples were available up to 90 days whereas development of Long COVID was defined as persisting symptoms 6–12 months after SARS-CoV-2 infection. Although our aim was to identify markers able to predict Long-COVID before it manifested itself, it would be of added value to monitor biomarker levels throughout the whole period of 12 months. Furthermore, sample size rapidly declined due to lack of follow-up after patients were released from the hospital, thereby losing statistical power.

In summary, our findings show that plasma levels for especially PTX3 and sMR are associated with COVID-19 disease outcome and survival. Furthermore, PTX3 and sMR concentrations were also linked to long-term immune recovery. Our results confirm previous studies showing that PTX3 is associated with COVID-19. Above all, we have identified sMR as novel biomarker that associates with COVID-19 disease course. Our findings imply that sMR has potential to serve as a marker for ICU admittance and prolonged survival following SARS-CoV-2 infection. Hence, alongside PTX3, measuring sMR levels would be of added value in monitoring COVID-19 disease course. Future studies are needed to confirm this set as COVID-19 biomarkers.

## STAR★Methods

### Key resources table

**Table:** 

REAGENT or RESOURCE	SOURCE	IDENTIFIER
**Biological samples**		

*COVID-19 cohort (EDTA plasma)*	*Addenbrooke’s Hospital*	N/A
*Additional samples from Healthy Controls*	*Semmelweis University Budapest*	N/A

**Critical commercial assays**		

Pentraxin 3, Human, ELISA Kit	Hycult Biotech	Cat# HK347
Q1q, Human, ELISA Kit	Hycult Biotech	Cat# HK356
C1-INH, Human, ELISA Kit	Hycult Biotech	Cat# HK396
C1s/C1-INH complex, Human, ELISA Kit	Hycult Biotech	Cat# HK399
Soluble mannose receptor, Human, ELISA Kit	Hycult Biotech	Cat# HK381
C3a, Human ELISA Kit	Hycult Biotech	Cat# HK354
C3c, Human ELISA Kit	Hycult Biotech	Cat# HK368
Soluble TCC, Human, ELISA Kit	Hycult Biotech	Cat# HK328
Human IL-6 Mag Bead Set	R&D systems/Biotechne	Cat#LHSCM206
Human IL-1b Mag Bead Set	R&D systems/Biotechne	Cat#LHSCM201
Human IL-10 Mag Bead Set	R&D systems/Biotechne	Cat#LHSCM217
Human TNF-a Mag Bead Set	R&D systems/Biotechne	Cat#LHSCM210
hIFN-g HS LxPA MAG	R&D systems/Biotechne	Cat#LHSCM285B
Base Kit, HS Cytokine A, Mag	R&D systems/Biotechne	Cat#LHSCM000

**Deposited data**		

Raw and analyzed data	This paper	This paper

**Software and algorithms**		

Graphpad Prism 10.1.2	Graphpad softwares Inc.	https://www.graphpad.com/scientific-software/prism/
BioRender	Science Suite Inc.	https://www.biorender.com
R statistical software	R Core Team, 2015	https://www.R-project.org/

**Other**		

Luminex	Bio-Plex, Bio-Rad, UK	N/A

### Resource availability

#### Lead contact

Further information and requests for resources and reagents should be directed to and will be fulfilled by the lead contact, Erik J.M. Toonen (e.toonen@hycultbiotech.com).

#### Materials availability

This study did not generate new unique reagents.

#### Data and code availability

• All data generated during the course of this study are included in the published article and supplemental information.
• This paper does not report original code.
• Any additional information required to reanalyze the data reported in this paper is available from the lead contact upon request.

### Experimental methods and study participant details

#### Ethics statement

Ethical approval for this study was obtained from the East of England – Cambridge Central Research Ethics Committee (‘‘NIHR BioResource’’ REC ref 17/EE/0025, and ‘‘Genetic variation AND Altered Leucocyte Function in health and disease - GANDALF’’ REC ref 08/H0308/176). Besides that, collection of additional healthy controls was approved by the Scientific and Research Ethics Committee of the Medical Research Council (ETT TUKEB) in Budapest, Hungary (8361-1/2011-EKU). All participants provided informed consent. Study was conducted in accordance with the Declaration of Helsinki.

#### Patient cohort

All study participants were recruited between 31st of March and 20th of July 2020 from patients attending Addenbrooke’s Hospital, Royal Papworth Hospital NHS Foundation Trust or Cambridge and Peterborough Foundation Trust, Cambridge, UK (cohort is previously described in detail by Bergamaschi and co-workers).^10^ One-hundred and fifty-nine COVID-19 patients with nucleic acid amplification test (NAAT) confirmed diagnosis and 56 PCR positive SARS-CoV-2 health care workers without symptoms or with mild symptoms (identified through routine staff screening) were enrolled. In total, 215 COVID-19 patients were included (mean age ± SD: 52·9 ± 17·8, male sex: 54·9%), together with 47 healthy controls (HC) (mean age ± SD: 42·3 ± 15·0, male sex: 55·3%). Controls were recruited among hospital staff attending Addenbrooke’s serology screening program and selected to cover the whole age spectrum of COVID-19 positive study participants, across both genders. Only controls with negative serology results were included in the study. Both patient and HC cohorts resemble the ethnic makeup of the UK, being 82% white and 18% Asian, Black, mixed or other ethnic group. Socioeconomic status of the participants was unknown. Analyses in ethnical subgroups were not performed due to limited sample size/lack of statistical power in these subgroups. For the sMR measurements, as limited sample volume from the healthy control group from Cambridge was available, additional healthy controls from Semmelweis University, Budapest, Hungary, were enrolled. The additional controls were sex and age-matched with controls from Cambridge.

Sampling was done at study entry, soon after infection or symptom onset (referred to as day 0), and then at regular intervals while patients remained admitted to hospital (approximately weekly up to 4 weeks). Discharged patients provided a follow-up sample 90 days after study enrolment. At each time-point, blood samples were drawn in EDTA blood collection tubes (BD Biosciences) and processed by the CITIID-NIHR COVID BioResource Collaboration group. Plasma samples were put on ice immediately after collection and processed within 1 h (centrifuged for 10 min., 2000x*g* at 4°C and stored at −80°C until further use). For the study presented here, plasma samples were available for the following timepoints: 0, 14, 28 and 90 days.

#### Clinical data collection

Clinical data were retrospectively collected by review of electronic medical charts (EPIC; EPIC System) and recorded in electronic case report forms (Castor EDC). As primary outcome disease severity was examined. COVID-19 patients were classified into two disease severity groups: moderate (asymptomatic, not requiring hospitalization (MILD) or hospitalized, but not requiring O_2_ or ventilation (HOSP)) and severe (hospitalized, requiring O_2_ (HOSP+O_2_) or admitted to intensive care unit (ICU)).

Long-term outcome, defined by immune recovery and the development of Long COVID, was examined as a secondary outcome. Immune recovery was classified as whether patients had persistently elevated (>10 mg/L) or recovering CRP (falling below 10 mg/L by final bleed), over 60 and 40 days respectively.^10^ Development of Long COVID was defined as persisting symptoms 6–12 months after SARS-CoV-2 infection. For this, ad hoc validated questionnaires for the assessment of long-term outcomes following COVID-19 were used. Questionnaires were based on a previously published tool for assessing rehabilitation need in patients who had prolonged ICU stays following COVID-19 infection.^80^ These questionnaires, assessing a range of long-term self-reported outcomes, were conducted in patients approximately 3–5 months and 9–10 months post symptom onset. Participants reported on symptoms arising, or worsening in severity, following SARS-CoV-2 infection and scores across seven symptom categories (fatigue, dyspnea, cough, pain, cognition and memory, new neurology, and muscle weakness) were used to classify individuals into “Long COVID” symptom groups. Study details are reported according to the RECORD statement (Data S1).

### Method details

#### Laboratory measurements

Plasma concentrations for the markers PTX3, C1q, C1-INH, C1s/C1-INH complex and sMR were measured in EDTA-plasma using commercially available ELISA kits (Cat#: HK347 (PTX3), HK356 (C1q), HK396 (C1-INH), HK399 (C1s/C1-INH), and HK381 (sMR), Hycult Biotech, Uden, The Netherlands). Measurements were conducted according to the manufacturers’ instructions. Biomarker concentrations were determined in duplicates, and measurements were performed in a blinded manner.

CRP, D-dimer and ferritin levels were measured by the Core Biochemical Assay Laboratory (CBAL) at Cambridge University Hospitals NHS Foundation Trust, using their standard assay. Serum levels of IL-1β, IL-6, IL-10, TNF-a and IFN-y were determined by the Clinical Immunology Laboratory at the Department of Biochemistry and Immunology of Addenbrooke’s Hospital Cambridge, using the high sensitivity Base Kit HS Cytokine A Mag (Cat# LHSCM000, R&D Systems) on a Luminex analyzer (Bio-Plex, Bio-Rad, UK). Laboratory values were previously reported.^10^

### Quantification and statistical analysis

As most of the continuous variables showed skewed distributions, data are presented as medians and interquartile ranges (IQR), and non-parametric statistical tests were used (Mann-Whitney U or Spearman rank test for comparison of two independent groups, Kruskal-Wallis test with Dunn’s post-test for multiple independent groups). A two-way ANOVA with Holm-Šídák’s multiple comparisons test was performed when analysing influence of surviving/disease severity/ICU admittance on biomarker levels over time. In the case of multiple comparisons, the level of significance was corrected by the Benjamini-Hochberg procedure to maintain a false discovery rate of 5%. Statistical calculations were performed with the GraphPad Prism 9 software (GraphPad Software Inc., La Jolla, CA, USA). Multivariate modelling was performed using Generalized Linear Models (GLM) implemented in the R statistical software (version 4.3.1). AIC (Akaike Information Criterion) was used to select the best predictor for disease severity, selecting the lowest AIC value as most suitable fit. A *p*-value < 0·05 was considered significant (∗*p* < 0·05, ∗∗*p* < 0·01, ∗∗∗*p* < 0·001, ∗∗∗∗*p* < 0·0001, ns = non-significant). Biomarker cut-off concentrations were determined according to the Youden’s index (J).^81^ A Youden’s index ≥50% was considered as suggestive of a biomarker having added value as a diagnostic test. Positive likelihood ratio (LR+) was calulated as sensitivity/(1-specificity), values > 1 increase the probability of the condition. Data is presented in violin plots with dashed horizontal lines indicating the median values and dotted lines indicating the quartiles.

## Consortia

Cambridge Institute of Therapeutic Immunology and Infectious Disease-National Institute of Health Research (CITIID-NIHR) COVID BioResource Collaboration

Stephen Baker, John R. Bradley, Patrick F. Chinnery, Daniel J. Cooper, Gordon Dougan, Ian G. Goodfellow, Ravindra K. Gupta, Nathalie Kingston, Paul J. Lehner, Paul A. Lyons, Nicholas J. Matheson, Caroline Saunders, Kenneth G. C. Smith, Charlotte Summers, James Thaventhiran, M. Estee Torok, Mark R. Toshner, Michael P. Weekes, Gisele Alvio, Sharon Baker, Areti Bermperi, Karen Brookes, Ashlea Bucke, Jo Calder, Laura Canna, Cherry Crucusio, Isabel Cruz, Rnalie de Jesus, Katie Dempsey, Giovanni Di Stephano, Jason Domingo, Anne Elmer, Julie Harris, Sarah Hewitt, Heather Jones, Sherly Jose, Jane Kennet, Yvonne King, Jenny Kourampa, Emily Li, Caroline McMahon, Anne Meadows, Vivien Mendoza, Criona O'Brien, Charmain Ocaya, Ciro Pascuale, Marlyn Perales, Jane Price, Rebecca Rastall, Carla Ribeiro, Jane Rowlands, Valentina Ruffolo, Hugo Tordesillas, Phoebe Vargas, Bensi Vergese, Laura Watson, Jieniean Worsley, Julie-Ann Zerrudo, Laura Bergamaschi, Ariana Betancourt, Georgie Bower, Ben Bullman, Chiara Cossetti, Aloka De Sa, Benjamin J. Dunore, Maddie Epping, Stuart Fawke, Stefan Gräf, Richard Grenfell, Andrew Hinch, Josh Hodgson, Christopher Huang, Oisin Huhn, Kelvin Hunter, Isobel Jarvis, Emma Jones, Maša Josipović, Ekaterina Legchenko, Daniel Lewis, Joe Marsden, Jennifer Martin, Federica Mescia, Francesca Nice, Ciara O'Donnell, Ommar Omarjee, Marianne Perera, Linda Pointon, Nicole Pond, Nathan Richoz, Nika Romashova, Natalia Savoinykh, Rahul Sharma, Joy Shih, Mateusz Strezlecki, Rachel Sutcliffe, Tobias Tilly, Zhen Tong, Carmen Treacy, Lori Turner, Jennifer Wood, Marta Wylot, John Allison, Heather Biggs, Helen Butcher, Daniela Caputo, Debbie Clapham-Riley, Eleanor Dewhurst, Christian Fernandez, Anita Furlong, Barbara Graves, Jennifer Gray, Tasmin Ivers, Emma Le Gresley, Rachel Linger, Mary Kasanicki, Sarah Meloy, Francesca Muldoon, Nigel Ovington, Sofia Papadia, Christopher J. Penkett, Isabel Phelan, Venkatesh Ranganath, Jennifer Sambrook, Katherine Schon, Hannah Stark, Kathleen E. Stirrups, Paul Townsend, Julie von Ziegenweidt, Jennifer Webster, Ali Asaripour, Lucy Mwaura, Caroline Patterson, Gary Polwarth, Katherine Bunclark, Michael Mackay, Alice Michael, Sabrina Rossi, Mayurun Selvan, Sarah Spencer, Cissy Yong, Petra Polgarova.
