# Supplementary material for: sMR and PTX3 levels associate with COVID-19 outcome and survival but not with Long COVID
Source: iScience. 2024 Jun 1;27(7):110162. doi: 10.1016/j.isci.2024.110162 (PMC11255846; doi:10.1016/j.isci.2024.110162)
Supplement: Document S1. Figures S1–S6, Table S1, and Data S1 [file mmc1.pdf]

iScience, Volume *27*

Supplemental information

sMR and PTX3 levels associate with COVID-19 outcome and survival but not with Long COVID

Lisa Hurler, Federica Mescia, Laura Bergamaschi, Cambridge Institute of Therapeutic Immunology and Infectious Disease-National Institute of Health Research (CITIID-NIHR) COVID BioResource Collaboration, Erika Kajdácsi, György Sinkovits, László Cervenak, Zoltán Prohászka, Paul A. Lyons, and Erik J.M. Toonen

Table S1. Laboratory values in moderate *vs.* severe COVID-19 patients (related to Figure 3)

Variables	Moderate COVID-19	Severe COVID-19	Moderate vs. severe
C3a (pg/mL)	94007 (68959-138602)	251169 (149917-403423)	***p*<0·0001**
C3c (ng/mL)	974 (686-1329)	1215 (919-1784)	***p*=0·0002**
sTCC (mAU/mL)	3013 (2318-3678)	4620 (3426-6503	***p*<0·0001**
hsCRP (ng/mL)	3·0 (1·2-12·1)	92·4 (27·3-202·6)	***p*<0·0001**
IL1β (pg/mL)	<0·06*	0·17 (0·1-0·3)	***p*<0·0001**
IL6 (pg/mL)	0·23 (0·19-0·70)	3·65 (1·79-12·19)	***p*<0·0001**
IL10 (pg/mL)	0·04 (0·04-0·29)	0·94 (0·29-1·61)	***p*<0·0001**
TNFα (pg/mL)	2·91 (2·08-4·59)	6·3 (3·96-9·69)	***p*<0·0001**
IFNγ (pg/mL)	<0·91*	0·91 (0·91-1·24)	*p*=0·1151
D-dimer (ng/mL)	142·0 (79·5-281·3)	747·5 (210·0-2427·0)	***p*<0·0001**
Ferritin (ng/mL)	162·3 (94·30-755·6)	732·0 (360·7-1262·0)	***p*=0·0006**

Biomarkers levels are depicted in median (IQR). *Assay detection limit

Figure S1. Age and BMI of healthy controls and moderate and severe COVID-19 patients (related to Figure 3). Distribution of age (a) and BMI (b) over the groups. No data regarding BMI was available for the controls.

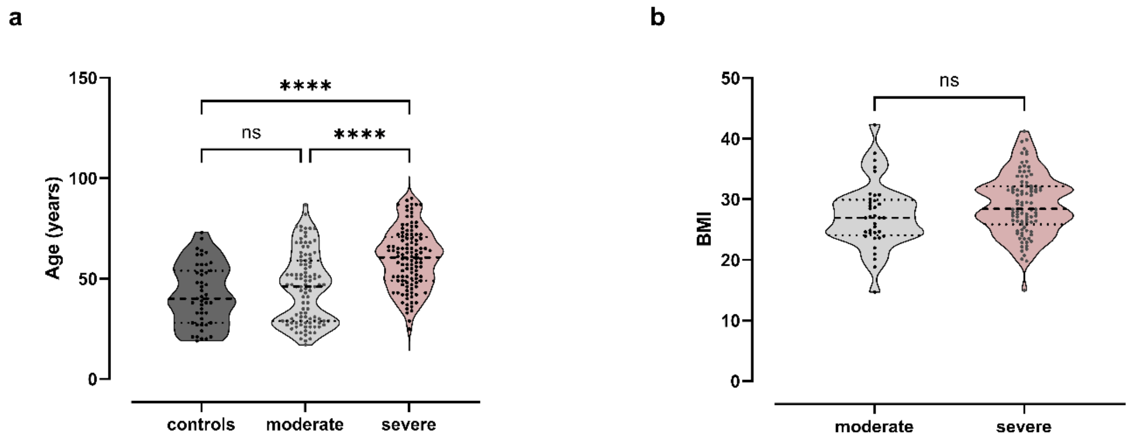

Figure S2. Laboratory values in healthy controls and in moderate and severe COVID-19 patients (related to Figure 3). (a) C3a, (b) C3c, (c) soluble TCC, (d) hsCRP, (e) IL1β, (f) IL6, (g) IL10, (h) TNFα, (i) IFNγ,(j) D-dimer, (k) Ferritin. For D-dimer and Ferritin, no levels in healthy controls were available.

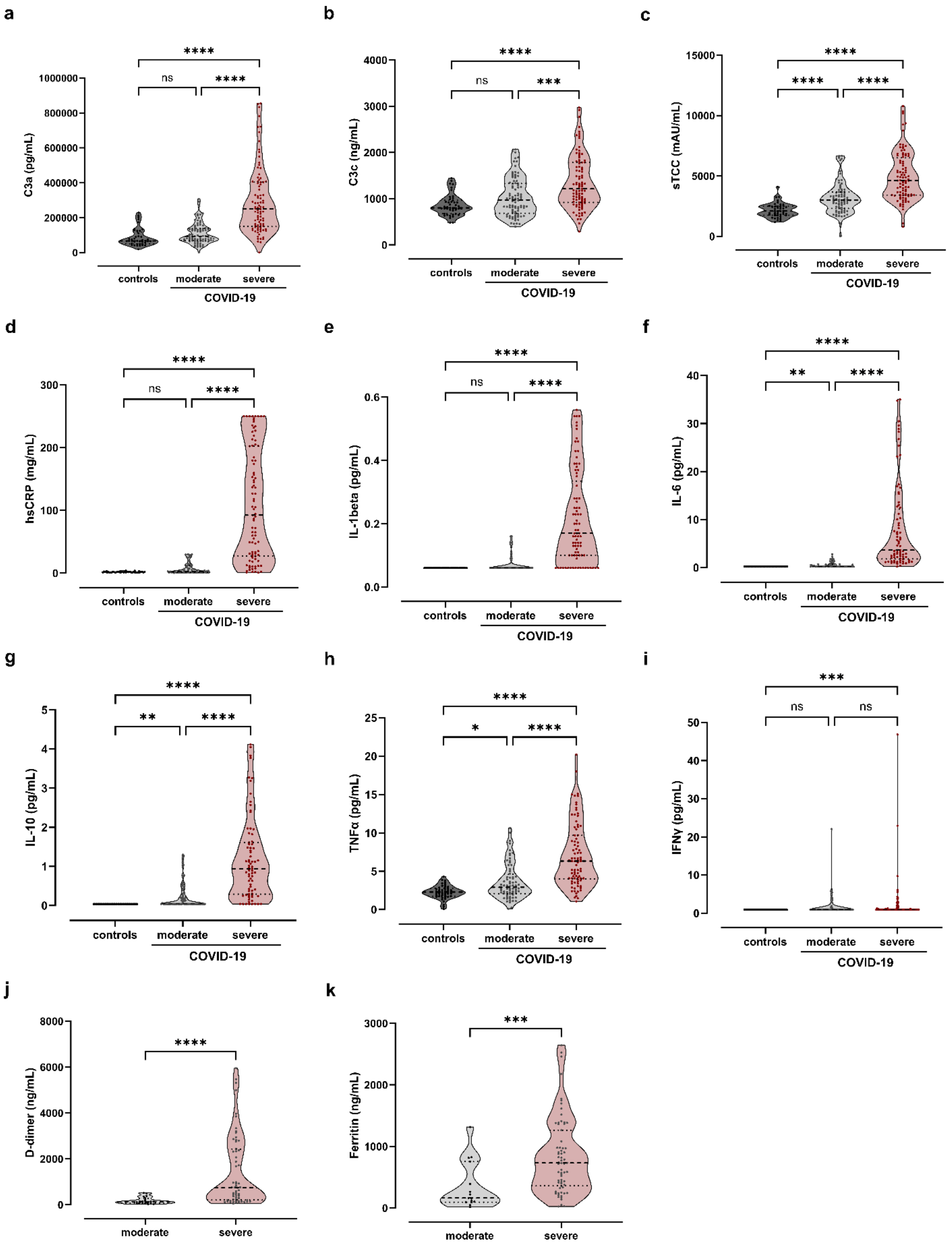

Figure S3. Correlation between laboratory and demographic parameters in COVID-19 patients (related to Figure 3). Heatmap of correlation matrix of laboratory and demographic parameters in COVID-19 patients. Color-coding indicates the strength of each correlation (Spearman correlation coefficients).

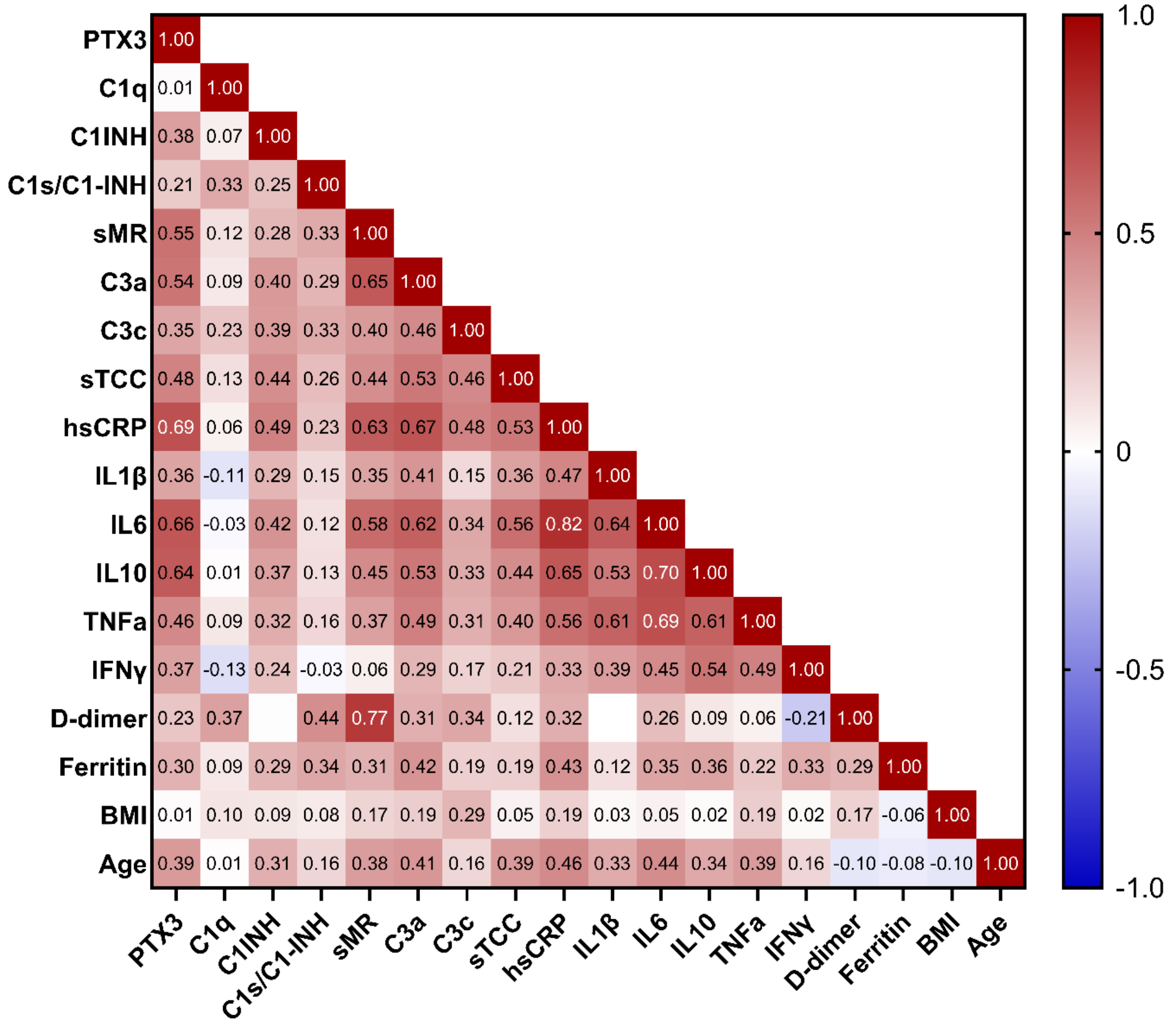

Figure S4. Different patterns of inflammation and activation markers between moderate and severe disease (related to Figure 3). Asterisks indicate significance comparing inflammation or activation marker levels (mean±SD) between moderate and severe COVID-19 patient groups at the given time points. Grey shaded area indicates biomarker concentration range in healthy controls.

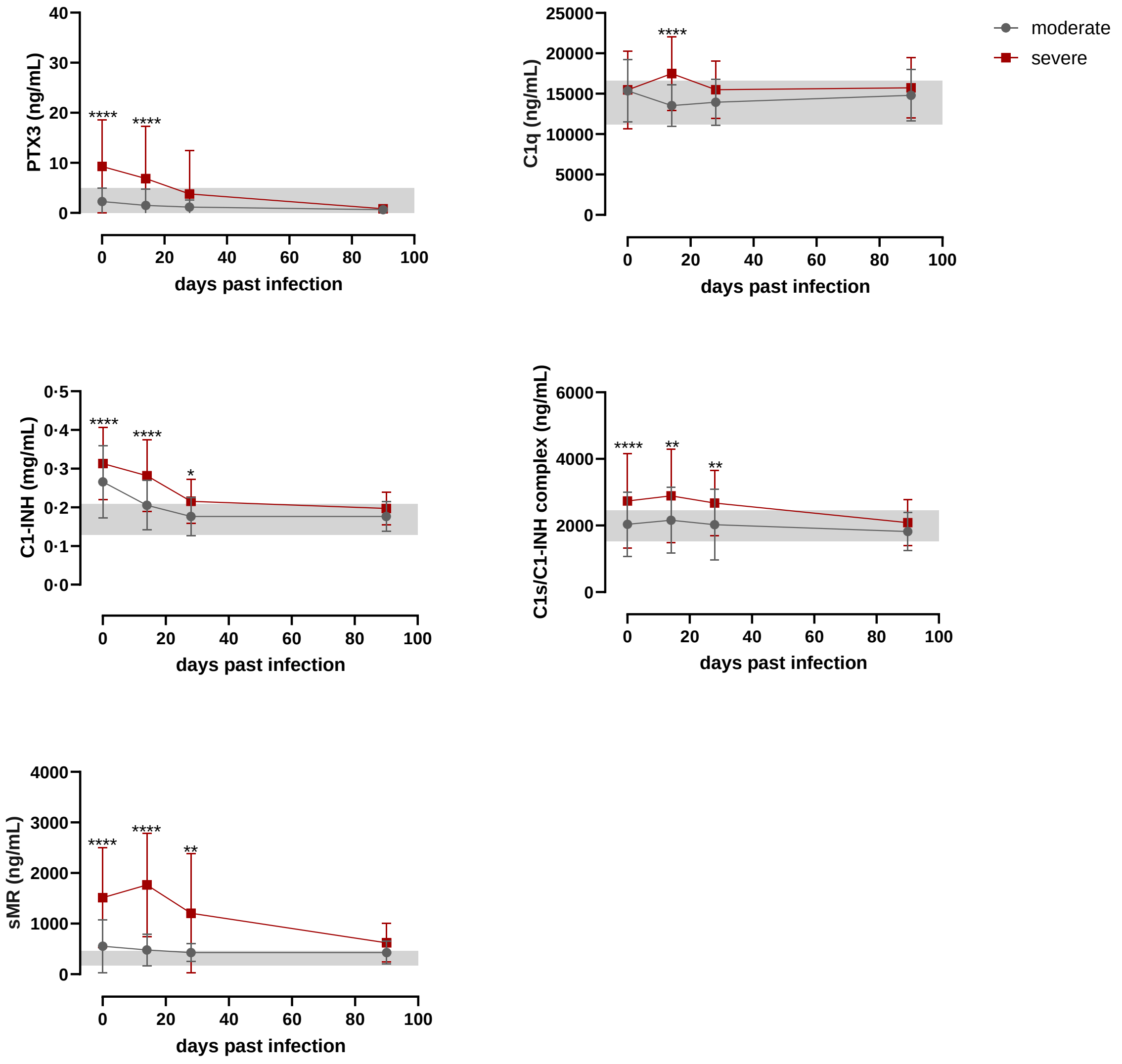

Figure S5. Different patterns of inflammation and activation markers between ICU and non-ICU patients in severe COVID-19 cases (related to Figure 4). Asterisks indicate significance comparing inflammation or activation marker levels (mean±SD) between non-ICU and ICU groups at the given time points. Grey shaded area indicates biomarker concentration range in healthy controls.

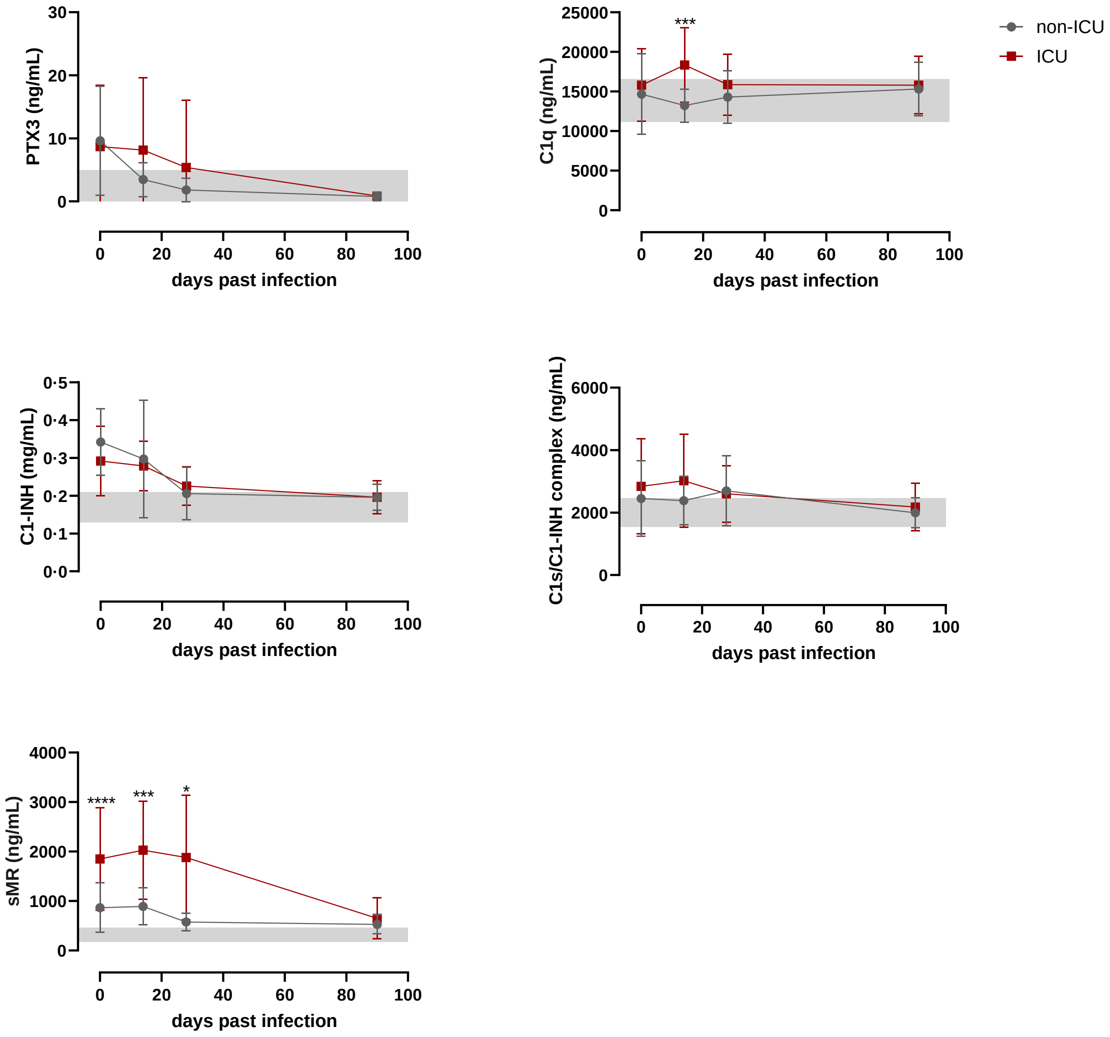

Figure S6. Different patterns of inflammation and activation markers between survivors and non-survivors in severe COVID-19 cases (related to Figure 5). Asterisks indicate significance comparing inflammation or activation marker levels (mean±SD) between survivors and non-survivors groups at the given time points. Grey shaded area indicates biomarker concentration range in healthy controls.

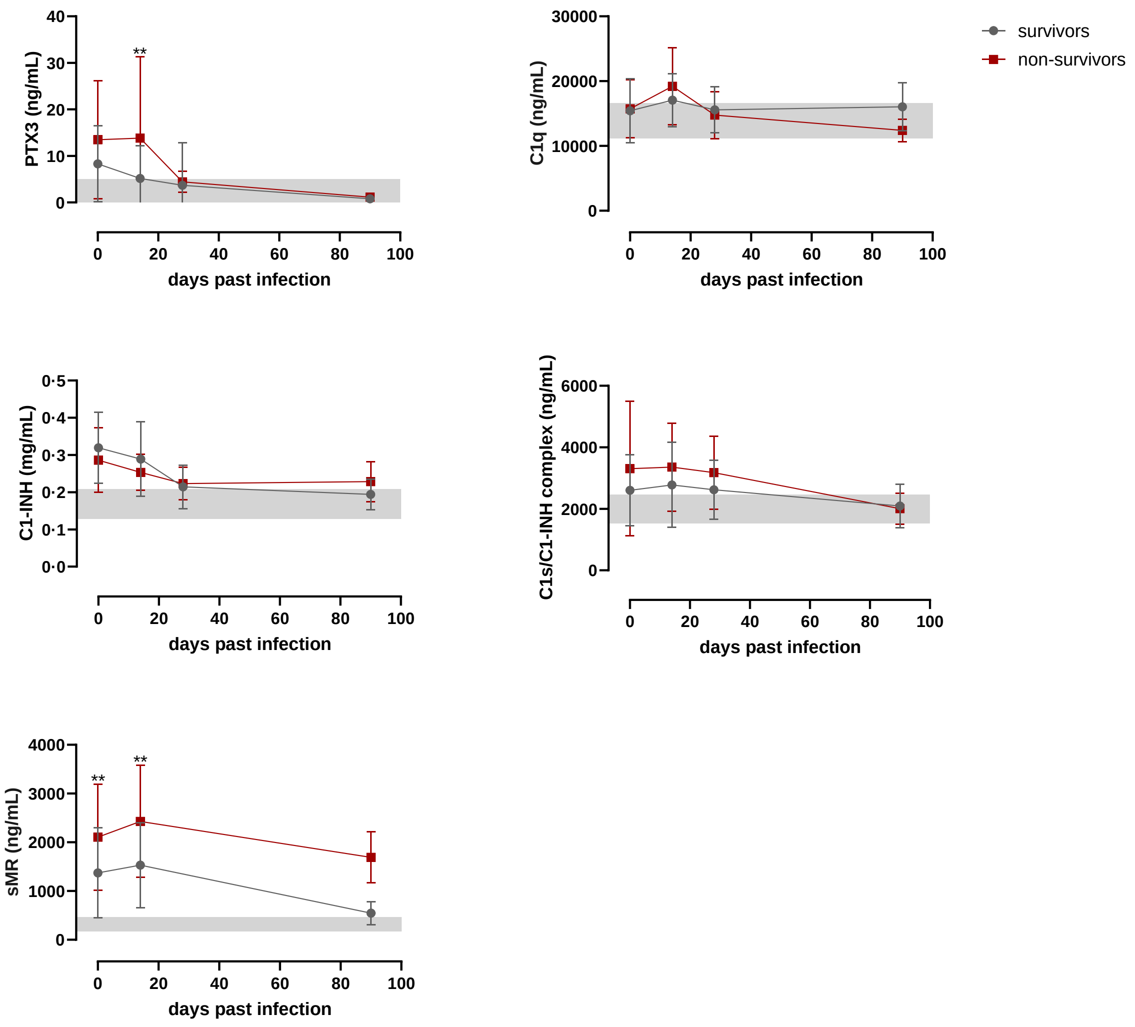

Data S1. The RECORD statement – checklist of items, extended from the STROBE statement, that should be reported in observational studies using routinely collected health data (related to STAR Metthodes).

	Item No.	STROBE items	Location in manuscript where items are reported	RECORD items	Location in manuscript where items are reported
**Title and abstract**					
	1	(a) Indicate the study's design with a commonly used term in the title or the abstract<br><br>(b) Provide in the abstract an informative and balanced summary of what was done and what was found	1<br><br>2	RECORD 1.1: The type of data used should be specified in the title or abstract. When possible, the name of the databases used should be included.<br><br>RECORD 1.2: If applicable, the geographic region and timeframe within which the study took place should be reported in the title or abstract.<br><br>RECORD 1.3: If linkage between databases was conducted for the study, this should be clearly stated in the title or abstract.	2<br><br>NA<br><br>NA
**Introduction**					
Background rationale	2	Explain the scientific background and rationale for the investigation being reported	3-4		
Objectives	3	State specific objectives, including any prespecified hypotheses	4		
**Methods**					
Study Design	4	Present key elements of study design early in the paper	27-30		
Setting	5	Describe the setting, locations, and relevant dates, including periods of recruitment, exposure, follow-up, and data collection	27-30		
Participants	6	*(a) Cohort study* - Give the eligibility criteria, and the	27-30	RECORD 6.1: The methods of study population selection (such as codes or	NA

		sources and methods of selection of participants. Describe methods of follow-up<br>*Case-control study* - Give the eligibility criteria, and the sources and methods of case ascertainment and control selection. Give the rationale for the choice of cases and controls<br>*Cross-sectional study* - Give the eligibility criteria, and the sources and methods of selection of participants<br><br>*(b) Cohort study* - For matched studies, give matching criteria and number of exposed and unexposed<br>*Case-control study* - For matched studies, give matching criteria and the number of controls per case	27-30<br><br>NA<br><br>NA<br><br>NA	algorithms used to identify subjects) should be listed in detail. If this is not possible, an explanation should be provided.<br><br>RECORD 6.2: Any validation studies of the codes or algorithms used to select the population should be referenced. If validation was conducted for this study and not published elsewhere, detailed methods and results should be provided.<br><br>RECORD 6.3: If the study involved linkage of databases, consider use of a flow diagram or other graphical display to demonstrate the data linkage process, including the number of individuals with linked data at each stage.	NA<br><br>NA
Variables	7	Clearly define all outcomes, exposures, predictors, potential confounders, and effect modifiers. Give diagnostic criteria, if applicable.	27-30	RECORD 7.1: A complete list of codes and algorithms used to classify exposures, outcomes, confounders, and effect modifiers should be provided. If these cannot be reported, an explanation should be provided.	NA
Data sources/ measurement	8	For each variable of interest, give sources of data and details of methods of assessment (measurement).<br>Describe comparability of assessment methods if there is more than one group	27-30		
Bias	9	Describe any efforts to address potential sources of bias	27-30		

Study size	10	Explain how the study size was arrived at	27-30		
Quantitative variables	11	Explain how quantitative variables were handled in the analyses. If applicable, describe which groupings were chosen, and why	27-30		
Statistical methods	12	(a) Describe all statistical methods, including those used to control for confounding<br>(b) Describe any methods used to examine subgroups and interactions<br>(c) Explain how missing data were addressed<br>(d) *Cohort study* - If applicable, explain how loss to follow-up was addressed<br>*Case-control study* - If applicable, explain how matching of cases and controls was addressed<br>*Cross-sectional study* - If applicable, describe analytical methods taking account of sampling strategy<br>(e) Describe any sensitivity analyses	29-30<br>29-30<br>27-30<br>27-30<br>27-30<br>NA		
Data access and cleaning methods		..		RECORD 12.1: Authors should describe the extent to which the investigators had access to the database population used to create the study population.<br><br>RECORD 12.2: Authors should provide information on the data cleaning methods used in the study.	27-30

Linkage		..		RECORD 12.3: State whether the study included person-level, institutional-level, or other data linkage across two or more databases. The methods of linkage and methods of linkage quality evaluation should be provided.	NA
**Results**					
Participants	13	(a) Report the numbers of individuals at each stage of the study (*e.g.*, numbers potentially eligible, examined for eligibility, confirmed eligible, included in the study, completing follow-up, and analysed)<br>(b) Give reasons for non-participation at each stage.<br>(c) Consider use of a flow diagram	5-9<br>NA<br>NA	RECORD 13.1: Describe in detail the selection of the persons included in the study (*i.e.*, study population selection) including filtering based on data quality, data availability and linkage. The selection of included persons can be described in the text and/or by means of the study flow diagram.	5-9
Descriptive data	14	(a) Give characteristics of study participants (*e.g.*, demographic, clinical, social) and information on exposures and potential confounders<br>(b) Indicate the number of participants with missing data for each variable of interest<br>(c) *Cohort study* - summarise follow-up time (*e.g.*, average and total amount)	5-9, 27-30<br>5-9<br>5-9		
Outcome data	15	*Cohort study* - Report numbers of outcome events or summary measures over time<br>*Case-control study* - Report numbers in each exposure category, or summary measures of exposure	5-9		

		Cross-sectional study - Report numbers of outcome events or summary measures			
Main results	16	(a) Give unadjusted estimates and, if applicable, confounder-adjusted estimates and their precision (e.g., 95% confidence interval). Make clear which confounders were adjusted for and why they were included<br>(b) Report category boundaries when continuous variables were categorized<br>(c) If relevant, consider translating estimates of relative risk into absolute risk for a meaningful time period	5-9<br><br>5-9<br><br>NA		
Other analyses	17	Report other analyses done—e.g., analyses of subgroups and interactions, and sensitivity analyses	5-9, 27-30		
**Discussion**					
Key results	18	Summarise key results with reference to study objectives	10		
Limitations	19	Discuss limitations of the study, taking into account sources of potential bias or imprecision. Discuss both direction and magnitude of any potential bias	13	RECORD 19.1: Discuss the implications of using data that were not created or collected to answer the specific research question(s). Include discussion of misclassification bias, unmeasured confounding, missing data, and changing eligibility over time, as they pertain to the study being reported.	10-14
Interpretation	20	Give a cautious overall interpretation of results considering objectives, limitations, multiplicity of analyses, results from similar	10-14		

		studies, and other relevant evidence			
Generalisability	21	Discuss the generalisability (external validity) of the study results	10-14		
**Other Information**					
Funding	22	Give the source of funding and the role of the funders for the present study and, if applicable, for the original study on which the present article is based	15		
Accessibility of protocol, raw data, and programming code		..		RECORD 22.1: Authors should provide information on how to access any supplemental information such as the study protocol, raw data, or programming code.	27-30
